# Esophageal Intraepithelial Neutrophil Infiltration is Common in Nigerian Patients With Non-Erosive Reflux Disease

**DOI:** 10.4021/gr284e

**Published:** 2011-01-20

**Authors:** Sylvester Chuks Nwokediuko, Uchenna Ijoma, Okechukwu Okafor

**Affiliations:** a Department of Medicine, University of Nigeria Teaching Hospital, Ituku/Ozalla, PMB 01129 Enugu, Nigeria; b Department of Morbid Anatomy, University of Nigeria Teaching Hospital, Ituku/Ozalla, PMB 01129 Enugu, Nigeria

**Keywords:** Intraepithelial, Neutrophil, Esophagus, NERD, Nigerians

## Abstract

**Background:**

Non-erosive reflux disease (NERD) is a variant of gastroesophageal reflux disease (GERD) in which patients with typical reflux symptoms have no evidence of erosive esophagitis at endoscopy. An objective diagnostic tool for NERD remains an unmet need for clinicians and researchers. This study was designed to determine the types of histological alterations seen in Nigerian patients with NERD.

**Methods:**

This was a prospective cross-sectional study in which mucosal biopsy was taken from the lower esophagus in patients with NERD. Similar biopsy was also taken from patients with nonulcer dyspepsia who served as controls. The materials were processed and examined histologically.

**Results:**

There were 68 patients with NERD and 60 patients with nonulcer dyspepsia. Intraepithelial neutrophil infiltration was significantly more frequent in patients with NERD compared to those with nonulcer dyspepsia (47.1% vs 13.3%, P = 0.0326). Epithelial proliferative chnges in the form of basal cell hyperplasia and papilla elongation were minimal (11.8% and 3.3% respectively).

**Conclusions:**

Nigerian patients with NERD have a high degree of esophageal intraepithelial neutrophil infiltration and a low prevalence of epithelial proliferative changes. This may be related to the relative rarity of Barrett’s esophagus in Nigerians.

## Introduction

Gastroesophageal reflux disease (GERD) is a condition that develops when the reflux of stomach contents causes troublesome symptoms with or without mucosal damage, and/or complications. Heartburn and regurgitation are typical symptoms of reflux experienced by patients [1, 2]. The prevalence of GERD is highest in North America and Europe, where at least weekly reflux symptoms range from 10 to 30%. Epidemiologic data are limited but suggest a lower prevalence in Asia [3], although prevalence is increasing in this region and other developed countries [4]. Gastroesophageal reflux disease was previously thought to be rare in Africans but recent studies actually indicate that it is common [5].

The disease adversely affects health-related quality of life [6], and the majority of patients (> 60%) with typical reflux symptoms have no evidence of erosive esophagitis at endoscopy [7, 8]. Such patients are usually considered to have non-erosive reflux disease or NERD [9].

Traditionally, GERD has been approached as a spectrum with NERD at the mild end and complicated GERD (stricture, Barrett’s esophagus or adenocarcinoma) at the other end of the spectrum. However recent data indicate that GERD may be categorized into three unique groups of patients or phenotypes: those with NERD, those with erosive esophagitis and those with Barrett’s esophagus [10].

The accurate assessment of NERD has proved difficult, as endoscopy does not provide any useful information, symptoms may be variable or atypical and even prolonged monitoring of esophageal pH shows no abnormality in about one-third of patients with otherwise typical symptoms [11]. Therefore, an objective diagnostic tool with acceptable sensitivity remains an unmet need for clinicians and researchers.

Over the years, various histological lesions have been described in patients with NERD. These studies were carried out in mainly Caucasian populations [12-14]. No such study has been done on Nigerians with NERD. Patients with NERD constitute over 60% of GERD in Nigeria [8]. This study was designed to determine the type and frequency of histological changes in the esophageal mucosa seen in Nigerian patients with NERD.

## Materials and Methods

This was a prospective cross-sectional study of patients with upper gastrointestinal symptoms seen at the gastroenterology unit of the University of Nigeria Teaching Hospital (UNTH) Ituku/Ozalla and Uzoma Specialist Hospital Trans Ekulu Enugu from June 2009 to December 2010. The study protocol was approved by the UNTH research ethics committee and informed consent was obtained from all the participants.

Patients who had heartburn and/or regurgitation were administered the Carlsson-Dent (CD) questionnaire [15]. This questionnaire utilized a symptom description and symptom analysis with numerical scores assigned to specific components of the symptom analysis. These scores could be positive or negative. When summed up, the total ranged from -7 to +18. The severity of symptoms was also graded from 1 to 5 representing no problem at all, mild problem, moderate problem, severe problem and very severe problem. The conditions for a diagnosis of GERD were a total score of 4 or higher in the CD questionnaire [15], and mild symptoms occurring on 2 or more days a week or more severe symptoms occurring at least once a week [2, 16]. Those diagnosed with GERD on this schema had standard upper gastrointestinal (GI) endoscopy and if no endoscopic lesion was found in the esophagus a diagnosis of NERD was made. Five mucosal biopsies were obtained from the lower esophageal area about 2 cm above the squamo-columnar junction (SCJ) around the 3 o’clock position.

Patients whose symptoms were epigastric pain, epigastric burning, postprandial fullness and/or early satiation were grouped as dyspepsia complex in accordance with Rome III guidelines [17, 18]. They also underwent upper GI endoscopy and those in whom no endoscopic lesion was found in the upper gastrointestinal tract (those with functional dyspepsia or non-ulcer dyspepsia) formed the control group. Biopsy of the lower esophageal mucosa was also obtained from them. Patients who had a combination of symptoms suggestive of GERD and dyspepsia were excluded from the study. Also excluded were patients who had hiatus hernia regardless of whether they had dyspeptic or reflux symptoms.

All the esophageal biopsy specimens were properly labeled, fixed in 10% buffered formalin, processed using paraffin embedding technique, sectioned at 4 micrometer perpendicular to the mucosal surface and stained with hematoxylin and eosin (H&E). Histological examination was performed by the same pathologist who was blind to the clinical diagnosis. The parameters scored were basal cell hyperplasia, papilla elongation, inflammation and dilated intercellular spaces. A score of 2 was regarded as the optimal cut-off value for separating GERD from non-GERD patients [19]. The results were expressed as means and proportions. Differences between means and proportions were determined and P values < 0.05 were considered statistically significant. A test of correlation was also carried out between the clinical criteria (score on CD questionnaire) and the histological criteria (reflux score) for the patients with NERD.

## Results

One hundred and twenty-eight (128) patients with upper GI symptoms participated in the study (68 patients with NERD and 60 patients with dyspepsia). There were 55 males (43.0%) and 73 females (57.0%). Table 1 illustrates the gender distribution of the patients. The mean age of patients with NERD was 51.8 ± 14.4 years while the mean age of the dyspeptic patients was 50.6 ± 14.8 years. The difference between the two means was not statistically significant (P = 0.9899). Table 2 illustrates the histological parameters. Dilated intercellular spaces (DIS) and intraepithelial neutrophil infiltration were the morphological changes that occurred more frequently in the patients with NERD compared to their dyspeptic counterparts. The difference was statistically significant (P = 0.0121 and 0.0326 respectively). Similarly, the mean reflux score in the patients with NERD was 2.29 ± 2.14 while the mean reflux score in the dyspeptic patients was 0.7 ± 0.7944. The difference was statistically significant (P = 0.0036). Using histological criteria for the diagnosis of GERD as proposed by Zentillin et al [19], 42 NERD patients (61.8%) qualified for a diagnosis of GERD while only 6 dyspeptic patients (10%) qualified for such diagnosis. The difference was statistically significant (P = 0.003).

**Table 1:** Gender Distribution of Patients With Upper Gastrointestinal Symptoms

Group	Male	Female	Total
NERD	28	40	68
Functional Dyspepsia	27	33	60
Total	55	73	128

**Table 2:** Histological Parameters (Esophageal Biopsy) in NERD and Dyspeptic Patients

Histological Parameter	NERD (n = 68)	Functional Dyspepsia (n = 60)	P value
Basal Cell Hyperplasia	8 (11.8%)	2 (3.3%)	0.2446
Papilla Elongation	8 (11.8%)	2 (3.3%)	0.2446
Dilated Intercellular Spaces	28 (41.2%)	4 (6.7%)	0.0121*
Eosinophils	4 (5.9%)	2 (3.3%)	0.6457
Neutrophils	32 (47.1%)	8 (13.3%)	0.0326*
Mean Score	2.29 ± 2.14	0.7 ± 0.7944	0.0036*
Score ≥ 2	42 (61.8%)	6 (10%)	0.003*

*Statistically significant

A positive correlation was demonstrated between the clinical and histological criteria [Pearson (γ) = 0.7767, P < 0.0001]. Thirty-two females had reflux scores ≥ 2 (43.8%), while only 10 males had reflux scores ≥ 2 (18.2%).

## Discussion

This study describes the histological changes in Nigerians with NERD using patients with functional dyspepsia as controls. Dilatation of intercellular spaces (DIS) was demonstrated in 41.2% of NERD patients compared to 6.7% of dyspeptic patients (P = 0.0121). It is one of the earliest changes resulting from acid injury to the esophageal epithelium and is the commonest morphological alteration seen in GERD. It has been proposed as a sensitive marker of acid-induced damage in the squamous epithelium. The description of this individual lesion, identifiable both by electron [20-22] and light microscopy [23, 24], has provided a stimulus for clinicians to reconsider histology in the diagnosis of GERD. It is not only more common in NERD patients compared to controls, but has also shown to improve after treatment with acid suppression [25]. However, there are concerns regarding the specificity of DIS as it is also found in association with psychological stress in animal models [26].

The remarkable finding in this study is the high prevalence of intraepithelial neutrophil infiltration in NERD patients. Inflammatory cells have previously been reported as rare in NERD [27]. Inflammation of any type (lymphocytes, eosinophils and neutrophils) had been adjudged to be more specific than sensitive for the diagnosis of reflux esophagitis [28, 29]. Neutrophils are the most abundant circulating leukocytes and they provide the first line of defense against tissue injury or infection. They release soluble chemotactic factors and proteases that alter the microenvironment and guide the recruitment of both nonspecific and specific immune effector cells [30]. Inflammatory and other immune cells undoubtedly take part in anti-tumor surveillance. In the absence of certain cells or functions, it is possible that some tumors will progress more rapidly [31-33]. Some studies have suggested that neutrophils are active in immunosurveillance against several tumors [34-36].

The histological changes in GERD generally and NERD specifically may be the forerunners of more serious complications such as Barrett’s esophagus. Barrett’s esophagus is known to exhibit racial variation, being more common in whites than blacks [37]. The significance of intraepithelial neutrophils is not clear but may be related to the relative rarity of Barrett’s esophagus in Nigerians and indeed blacks. Furthermore, changes of epithelial proliferation (basal cell hyperplasia and papilla elongation) were not common in NERD patients in this study. However, some studies have also shown that tumor-associated monocytes/macrophages are essential promoters of tumor cell migration, invasion and metastasis [38]. Inflammation is now considered a well-established cancer risk factor: a number of inflammatory conditions predispose to cancer, including ulcerative colitis and Barrett’s esophagus [39]. So it is not surprising that the regular use of aspirin and other non-steroidal anti-inflammatory drugs (NSAIDs) is related to a decreased risk of several types of cancer. Compelling data from epidemiological studies, intervention trials and animal studies indicate that aspirin and other NSAIDs inhibit colorectal carcinogenesis [40, 41]. Epidemiological evidence is accumulating that aspirin or NSAID use is protective against esophageal and gastric cancer, and possibly also against cancers of prostate, ovary and lung [42-47]. More studies, including genetic studies, are clearly needed not only to further elucidate the significance of intraepithelial neutrophil infiltration in NERD but also to determine the benefits and deleterious effects of inflammation in carcinogenesis.

There were more females (57%) than males (43%) in the population studied. Similarly, the relative proportion of females who had a reflux score (histology) of ≥ 2 was higher than the relative proportion of males (43.8% versus 18.2%). This is consistent with findings from other studies across the globe. There is a consistent female preponderance in dyspepsia [48-52] and patients with NERD often have other functional gastrointestinal symptoms, such as functional dyspepsia and irritable bowel syndrome (IBS), with a frequency higher than observed in most studies of erosive reflux disease [53-55].

In conclusion we have demonstrated that Nigerian patients with NERD have a high prevalence of esophageal intraepithelial neutrophil infiltration and a low prevalence of epithelial proliferative changes. It remains to be shown whether these changes are related to the observed relative rarity of Barrett’s esophagus in Nigerian patients with GERD.
